# Lesion-Aware Multiple-Instance Learning for Grapevine Disease Recognition from Field RGB Images

**DOI:** 10.3390/s26196318

**Published:** 2026-10-07

**Authors:** Övünç Öztürk, Bora Canbula, Tuğba Özacar, Vehbi Özacar

**Affiliations:** 1 Department of Computer Engineering, Manisa Celal Bayar University, Şehit Prof. Dr. İlhan Varank Campus, Manisa 45140, Türkiye; bora.canbula@cbu.edu.tr (B.C.); tugba.ozacar@cbu.edu.tr (T.Ö.); 2 Octahedron Innovation Informatics Electronics and Mining Services Inc., Manisa Technopark, Manisa 45140, Türkiye; vehbi.ozacar@deu.edu.tr; 3 Canbula Software Research and Development Ltd. Co., Manisa Technopark, Manisa 45140, Türkiye; 4 Torbalı Vocational School, Dokuz Eylül University, İzmir 35860, Türkiye

**Keywords:** grapevine disease recognition, multiple-instance learning, annotation-guided learning, offline image analysis, bootstrap uncertainty, ablation study, lesion detection

## Abstract

This retrospective offline study examines how dense manual regional annotations can be organized for image-level grapevine disease recognition using pre-acquired HERMOS RGB images. After audit and filtering, 491 source images and 13,765 Pascal VOC regions were retained. A leakage-safe source-image split was used across full-image classification, regional crop classification, crop-to-image aggregation, annotation-guided multiple-instance learning (MIL), and YOLO11 localization. MIL bags in training, validation, and testing were constructed from crops derived from manual boxes; the results therefore describe annotation-assisted aggregation rather than raw-image deployment. The original ConvNeXt-Tiny MIL model achieved macro-F1 0.922 (95% source-image bootstrap CI 0.882–0.956), compared with 0.836 (0.754–0.895) for full-image ConvNeXt-Tiny and 0.869 (0.804–0.917) for the best fixed crop aggregation. Paired macro-F1 differences favored MIL over these baselines by 0.087 (95% CI 0.029–0.163; Holm-adjusted *p* = 0.013) and 0.053 (0.005–0.114; adjusted *p* = 0.036), respectively. Downy mildew precision and recall were 1.000 on nine positive test images, but the corresponding exact 95% binomial intervals were 0.664–1.000. Controlled ablations produced lower point estimates without learned attention or the auxiliary instance loss, whereas removing context expansion did not reduce performance; none of the three paired ablation tests was significant after Holm correction. Increasing detector capacity from YOLO11n to YOLO11s did not improve full-image mAP@0.50 (0.175 versus 0.170). These findings support annotation-guided local-evidence aggregation as an offline exploratory benchmark, while not establishing split-to-split stability, computational efficiency, or external-vineyard generalization.

## 1. Introduction

Grapevine diseases can reduce canopy function, yield, fruit quality, and the efficiency of crop-protection programs. RGB imagery provides an accessible visual record for retrospective disease analysis, but reliable decisions remain difficult because background clutter, illumination, symptom similarity, and lesion scale can alter the visible signal [1,2]. Deep convolutional networks established that plant diseases can be recognized directly from leaf images [3,4], and field-oriented studies later demonstrated the importance of acquisition conditions outside controlled laboratories [5]. The present work is a retrospective offline analysis of pre-acquired close-up HERMOS images. It does not evaluate camera hardware, real-time inference, mobile or edge execution, computational efficiency, operational screening, or external-vineyard generalization. Transfer to canopy-scale imagery would require an upstream leaf or candidate-region stage and separate validation under scale, occlusion, and background shift.

Grapevine research has followed several complementary directions. Real-time detectors have been developed for grape leaf diseases [6], while recent studies have emphasized lightweight edge classification [7], transformer-enhanced detection [8], UAV-based recognition [9], improved residual networks [10], attention-guided lightweight models [11], and explainable compact networks [12]. These advances show the rapid development of the field, but their reported scores are not directly comparable because datasets, disease taxonomies, image conditions, annotation types, and split protocols differ.

The visual evidence is also disease dependent. Powdery mildew can appear as localized surface changes, downy mildew as patch-like discoloration, and diseases associated with grapevine trunk disorders as heterogeneous necrotic or discoloration patterns [13,14,15]. A single source image may therefore contain healthy tissue together with several small symptomatic regions. This structure makes the representation of local evidence as important as the choice of backbone architecture.

The HERMOS dataset is particularly useful for studying this question because it combines field-oriented grapevine images with dense Pascal VOC bounding boxes for healthy tissue, powdery mildew, dead arm, and downy mildew [16]. The boxes can be treated as independent crop labels, strict detection targets, or local instances that support an image-level decision. Each formulation answers a different question and can produce different failure modes.

This study evaluates five formulations under one leakage-safe source-image split: full-image multi-label classification, crop classification, crop-to-image aggregation, annotation-guided attention-based MIL constructed from manually annotated regions, and YOLO11 lesion detection. The contribution is a comparative offline pattern-recognition analysis of how dense regional annotations can be operationalized. To quantify fixed-test-set uncertainty and examine component-specific hypotheses, the analysis additionally includes source-image bootstrap intervals, paired statistical tests, controlled MIL ablations, and a YOLO11s capacity control. The MIL branch is not presented as a raw-image end-to-end system because its training, validation, and test bags all depend on manual HERMOS boxes. Accordingly, the results are interpreted as an annotation-assisted aggregation benchmark, attention is treated as qualitative model evidence, and detector conclusions are limited to the evaluated YOLO11n and YOLO11s configurations.

The specific contributions are:

(1) A common source-image protocol that prevents leakage at the crop level across all learning formulations;
(2) An annotation-guided gated-attention MIL formulation that combines bag-level disease-presence targets with box-derived regional supervision;
(3) Source-image bootstrap uncertainty estimates and paired tests for the primary image-level comparisons, together with controlled ablations of attention, context expansion, and the auxiliary instance loss;
(4) An exact archived-configuration audit and a YOLO11s capacity control, reported within a strictly offline exploratory scope.

## 2. Related Work and Research Gap

This section positions the study within field-oriented plant and grapevine disease recognition, multiple-instance learning, visual backbones, detection, and model evidence methods. The review focuses on differences in supervision, prediction unit, and use of regional annotations, and then defines the specific gap addressed by the common source image comparison.

Field-oriented plant and grapevine disease studies are particularly relevant to the present work because they address the challenges of recognizing disease symptoms under natural imaging conditions, including background clutter, illumination variation, scale changes, and occlusion. Controlled datasets such as PlantVillage have been important for establishing the feasibility of image-based diagnosis, whereas PlantDoc and other natural-image datasets exposed the harder conditions created by clutter, occlusion, scale variation, and illumination changes [17,18]. Region-aware plant disease analysis has taken several forms. Lesion-level classification evaluates the diagnostic value of individual spots [19]; detector-based studies use box-defined candidate regions [6,8,20]; image-level and multimodal systems predict one or more disease states from an entire image [21,22]; and few-shot methods address adaptation under limited field data [23]. These lines of work differ in supervision, prediction unit, and operational objective. Most grapevine studies optimize one formulation at a time, which makes it difficult to separate the effect of the backbone from the effect of annotation representation and aggregation.

A second relevant line of work is multiple-instance learning, which provides a natural framework for aggregating local evidence. MIL attaches supervision to a bag of instances rather than requiring a definitive label for every instance [24,25,26]. In bag-label-only settings, it is commonly described as weakly supervised. Attention-based deep MIL learns differentiable weights that summarize the contribution of each local instance to the bag-level prediction [27]. The approach has been particularly influential in computational pathology, where large images are divided into patches and the final diagnosis is made at slide level [28,29]. The present study differs from a conventional bag-label-only MIL because manual box labels are also used by an auxiliary instance-level objective; the experimental formulation is therefore described as annotation-guided or hybrid-supervision MIL. Transformer-based MIL subsequently modeled relationships among correlated instances [30]. More recent pathology foundation models, including UNI and CONCH, demonstrate how strong pretrained visual or vision-language encoders can support patch-based aggregation [31,32]. These methods motivate future transformer and foundation-model baselines for HERMOS, but they were not included in the evaluated experiment set.

Beyond the aggregation strategy, the choice of visual backbone, localization framework, and model-evidence method also shapes performance and interpretation. The classification experiments compared ResNet50, EfficientNet-B0, and ConvNeXt-Tiny [33,34,35]. These networks represent residual learning, compound scaling, and a modernized convolutional design influenced by vision transformers [36]. The comparison therefore tests whether the same regional representation behaves consistently across distinct encoder families.

Bounding box annotations also motivate explicit localization. Pascal VOC established a standard object-detection evaluation setting [37], and YOLO formulated detection as a unified prediction problem [38]. Detector and region-proposal pipelines can create local candidate regions automatically, whereas the present MIL branch uses the existing manual HERMOS boxes. The experiments use YOLO11n as the primary compact baseline and YOLO11s as a higher-capacity control; conclusions are therefore limited to these two configurations [39]. Attention maps and related attribution methods can be useful to inspect model evidence [40,41,42], but attention weights are not causal explanations and should not be equated with quantitative localization fidelity. Sensor-oriented plant-disease research has also addressed real-time detection [20], multimodal interpretation [21], few-shot field adaptation [23], and acquisition-aware image sensing [43]. These studies provide context, whereas the present work remains a retrospective offline analysis of pre-acquired images.

Taken together, these studies suggest that an important question remains regarding how pre-existing regional annotations can be used effectively for source-image diagnosis when multiple small regions coexist. This comparison does not test automatic region discovery. A full-image model may dilute small lesions during resizing and pooling. A crop classifier recognizes local appearance but requires a fixed aggregation rule. A detector must localize every region sufficiently well to satisfy box-level metrics. Annotation-guided MIL retains the manual local regions while optimizing the image-level label directly. A fair comparison requires a common source-image split so that crops from the same leaf cannot cross data partitions. Because the MIL bags are built from manual boxes in every partition, its result is an annotation-assisted offline aggregation benchmark and should not be interpreted as deployable raw-image performance.

## 3. Materials and Methods

This section describes the dataset audit, leakage-safe partitioning, derived image representations, classification and MIL formulations, YOLO11 localization baselines, exact archived implementation settings, and the fixed-split statistical analysis. Particular attention is given to annotation-assisted MIL inference and to separating test-sample uncertainty from split-to-split or training-seed stability.

### 3.1. Dataset Audit and Image-Level Target

The study used the HERMOS grapevine leaf disease dataset [16]. The local audit identified 492 image files and 492 XML annotation files. After canonical label mapping and invalid box filtering, 13,765 usable objects remained from 13,904 parsed objects. One image without a usable canonical object was excluded, leaving 491 source images. The retained regional classes were healthy, powdery mildew, dead arm, and downy mildew. The term “images with >1 regional class” is used below for images containing more than one of these four canonical regional classes. It is not equivalent to multiple image-level disease presence labels, because healthy and diseased regions may coexist within the same source image. The resulting audit is summarized in Table 1.

The primary target at the image level represented the presence of three diseases: powdery mildew, dead arm, and downy mildew. Healthy annotations were retained for regional crop classification because they identify healthy local tissue; they were not treated as an independent positive disease label at image level. This distinction prevents a healthy crop inside a partly diseased image from being interpreted as a healthy source image.

Table 2 places HERMOS beside widely used plant disease resources. PlantVillage is much larger but is dominated by controlled acquisition and labels assigned at the image level. PlantDoc provides natural-image object annotations across multiple crops. HERMOS is smaller, grapevine specific, and unusually dense at the regional level, which makes it suitable for studying local evidence aggregation rather than large-scale category coverage. The comparison was compiled from the cited sources; the HERMOS counts reflect the local audit and filtering reported in Table 1.

### 3.2. Leakage-Safe Split and Derived Representations

The source image was the fundamental split unit. The final partition contained 344 training images, 74 validation images, and 73 test images. Every full image, crop, context crop, MIL, and detection view inherited the same assignment. Consequently, visually related crops from one leaf could not appear in both training and testing. The 212 downy mildew objects in the test partition are local boxes clustered within only nine positive source images, so object count and support at the image level represent different units. The split and regional object counts are reported in Table 3.

Four image representations were derived. The full-image view paired each source image with a three-label disease vector. The bounding-box view treated each valid manual annotation as a four-class regional crop. The context crop was a square centered on the manual box with a side length of 1.75 × max (box width, box height); lower coordinates were rounded with floor, upper coordinates with ceil, and the result was clipped to the image boundaries without padding. Bounding-box and context crops were exported as 150 × 150 JPEG images (quality 95, Lanczos resampling) and resized to 224 × 224 with bilinear interpolation for crop and MIL models. The MIL view treated all manual annotation-derived crops from one source image as a bag in training, validation, and testing; no automatic proposal stage was used. The detector branch used either full images or 1280 × 1280 tiles with 25% overlap (960-pixel stride), a minimum visible-box fraction of 0.25, and a minimum clipped box width and height of 4 pixels. Tile predictions were evaluated as independent detector samples and were not remapped and merged at source-image level. Table 4 summarizes the learning formulations and evaluation targets.

**Figure 1:**
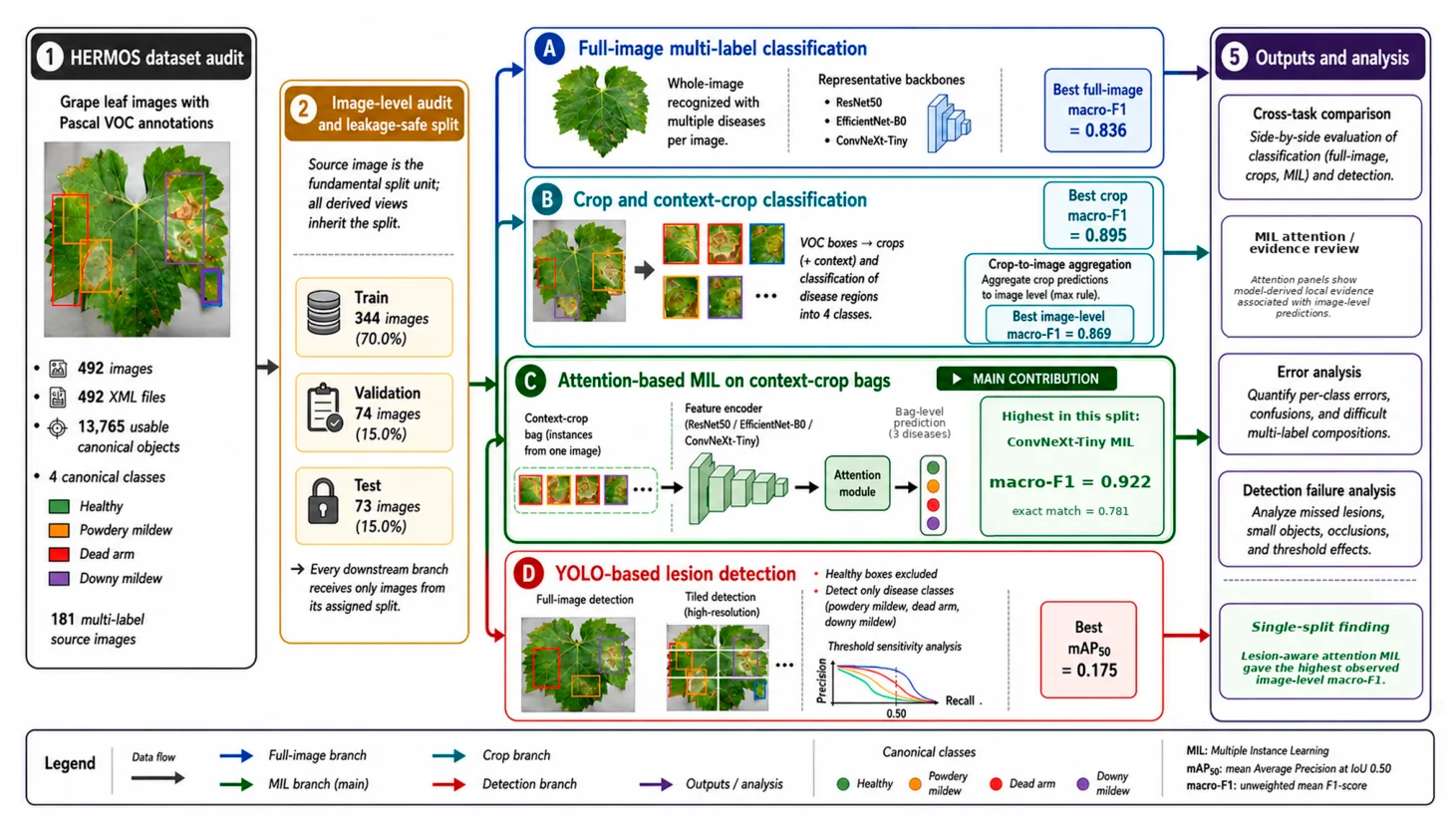
The common source image split and the five derived learning formulations.

Figure 1 summarizes the common source-image split and the five core learning formulations. All representations inherit the same split, while the MIL branch uses manually annotated crops in every partition and is therefore annotation-assisted. The source-image bootstrap analysis, paired tests, component ablations, and YOLO11s capacity control operate on the same split assignments and are reported in Section 3.7 and Section 4.3, Section 4.4, Section 4.5 and Section 4.6. Attention is not interpreted as a causal or disease-specific explanation.

### 3.3. Classification and Crop-Aggregation Baselines

ResNet50, EfficientNet-B0, and ConvNeXt-Tiny were evaluated as ImageNet-pretrained visual encoders [33,34,35]. Full-image models produced three sigmoid probabilities. Regional crop models produced a four-class softmax prediction, and crop-to-image aggregation took the maximum predicted probability for each disease across all crops belonging to the same source image. This fixed rule is sensitive to sparse positive evidence but can also amplify an isolated high-probability false positive. Exact preprocessing, optimization, class-weighting, checkpoint, and threshold-selection settings are reported in Table 5.

The validation partition was used exclusively for checkpoint and operating-point selection, and the test partition was reserved for final reporting. Full-image and image-aggregated crop thresholds were selected independently for each disease by a 0.01-step validation grid search that maximized class-wise F1, then frozen for the test set. The primary reported thresholds were [0.53, 0.44, 0.22] for full-image ConvNeXt-Tiny and [0.40, 0.89, 0.90] for context-crop ResNet50 maximum aggregation, in the order of powdery mildew, dead arm, and downy mildew, respectively.

### 3.4. Annotation-Guided MIL Formulation

For source image *i*, the annotation-guided MIL bag *B_i_* = {*x*_*i*1_, …, *x_in_i__*} contains context crop instances derived from that image’s manually annotated boxes. A shared encoder *f_theta_* maps each instance to an embedding *h_ij_*. Gated attention assigns a normalized weight to each embedding and forms a bag descriptor *z_i_* [27]. The gated attention module produces one shared distribution over instances for the entire bag; it is not disease-specific. In editable-text form, the aggregation is:

*a_ij_* = *softmax_j_*{*w^T^* [*tanh*(*V h_ij_*) ⊙ *sigmoid*(*U h_ij_*)]}, *z_i_* = *Σ_j_ a_ij_ h_ij_*. (1)

A bag classifier maps *z_i_* to three disease probabilities for powdery mildew, dead arm, and downy mildew. The bag target is the three-label disease-presence vector. The auxiliary instance target is the four-class regional label inherited from each manual annotation: healthy, powdery mildew, dead arm, or downy mildew. Because the instance objective uses box-derived labels, the formulation is annotation-guided or hybrid-supervision MIL rather than conventional bag-label-only weak supervision. The combined objective is:

*L* = *L_bag_* + *λ L_instance_*. (2)

Here, *L_bag_* is a three-output binary-cross-entropy-with-logits loss with positive weights [1.000, 1.774, 7.390], *L_instance_* is a four-class cross-entropy loss with class weights [0.681, 0.634, 1.847, 2.425], and *lambda* = 0.2 in the full model. The bag task remains primary. Gated attention uses a 256-dimensional hidden layer and a dropout of 0.2 and produces one shared distribution over instances for all three output labels; consequently, a high instance weight cannot be assigned uniquely to one disease in a multi-label image.

Before the component ablations, the archived full MIL configuration was rerun. A macro-F1 difference within ±0.02 of the original result was specified as the reproduction gate. The three pre-specified ablations then changed one principal component at a time relative to this contemporaneous reference: uniform mean pooling replaced learned gated attention; original bounding-box crops replaced 1.75× context crops while retaining the same encoder initialization and remaining settings; or the auxiliary loss was removed by setting *lambda* = 0. All ablations used the same source-image split and seed 52.

### 3.5. YOLO11 Localization Baselines

The detection branch used YOLO11n as the primary compact strict-localization baseline and YOLO11s as a higher-capacity full-image control [39]. Healthy boxes were excluded, and the class order was 0: powdery mildew, 1: dead arm, and 2: downy mildew. The two full-image models used the same dataset, split, initialization convention, 1280-pixel input, training schedule, and test protocol; the tiled experiment was performed with YOLO11n.

Pascal VOC boxes were converted to normalized YOLO center/width/height coordinates. For image width *W*, image height *H*, and *box* (*x_min_*, *y_min_*, *x_max_*, *y_max_*), *x_c_* = (*x_min_* + *x_max_*)/(2*W*), *y_c_* = (*y_min_* + *y_max_*)/(2*H*), *w* = (*x_max_* − *x_min_*)/*W*, and *h* = (*y_max_* − *y_min_*)/*H*. Invalid boxes were removed during the common audit. Detector validation used *IoU* = 0.70 for non-maximum suppression, *max_det_* = 300, and class-aware NMS. Ultralytics best.pt was selected by validation fitness (mAP@0.50:0.95 in the archived runs) with patience 15. Exact training and augmentation values are listed in Table 5.

In addition to standard detector metrics, a fixed-confidence threshold sweep was evaluated for YOLO11n as a descriptive post hoc analysis. It was not used to train weights or select an independently validated operating point. The reported thresholds were 0.10–0.15 for full images and 0.08–0.15 for tiled images. Precision, recall, and micro-F1 are reported for these thresholds; “highest observed” refers only to the included sweep.

### 3.6. Implementation Details and Hyperparameters

Table 5 presents an audited summary of the exact implementation and analysis settings, run configurations, class-weight files, threshold files, detector arguments, and run summaries. The same settings were used for the full-model reproduction and ablation runs unless the ablated component is explicitly identified.

For additional model context, Table 6 reports the post-resizing input tensor shape and the exact total number of trainable parameters for every evaluated task-specific architecture. Counts were obtained directly from the instantiated experimental models by summing parameters with requires_grad=True and therefore include the corresponding task-specific heads.

### 3.7. Evaluation and Statistical Analysis

Models operating at the image level were evaluated with macro-F1, micro-F1, exact-match accuracy, macro-AUROC, and macro average precision. Macro-F1 was the pre-specified primary metric because it gives equal weight to the three disease classes. Crop models were evaluated with crop macro-F1, and aggregated predictions were evaluated with image-level metrics. Detection was evaluated with precision, recall, mAP@0.50, and mAP@0.50:0.95; metrics defined on different prediction units are not directly interchangeable. Fixed trained models were evaluated by 10,000 source-image bootstrap resamples of the 73-image test set. Percentile 95% intervals were calculated for model metrics and paired model differences. Paired prediction-swap randomization tests were used for macro- and micro-F1, exact McNemar tests for exact match, and Holm correction for the two primary baseline macro-F1 comparisons. For the three pre-specified ablation macro-F1 tests, Holm correction was applied as a separate family. Exact Clopper-Pearson intervals were used for downy-mildew precision and recall because ordinary bootstrap intervals degenerate when all nine positives are classified correctly.

All values come from one leakage-safe train/validation/test partition. The bootstrap intervals quantify test-sample uncertainty for fixed trained models under this partition; they do not establish training-seed or split-to-split stability. Performance stability across alternative random data splits remains unverified because the models were not retrained under alternative split assignments. The paired tests likewise compare predictions on the same 73 source images. Model input shapes and exact trainable parameter counts are reported in Table 6 solely to provide architectural context. Crop-level and image-level values remain separated by prediction unit, attention panels remain qualitative, and no latency, FLOPs, memory, mobile-device, or external-vineyard result is used to support deployment claims.

## 4. Results

This section reports fixed-split results using the prediction unit appropriate to each task. It summarizes the original model results, source-image uncertainty and paired comparisons, controlled MIL ablations, bag-level errors and attention patterns, and YOLO11 detector capacity control.

### 4.1. Cross-Task Summary

The audited dataset contained 13,765 usable regions. Full-image ConvNeXt-Tiny reached macro-F1 0.836, context-crop ConvNeXt-Tiny reached crop macro-F1 0.895, context-crop ResNet50 maximum aggregation reached image-level macro-F1 0.869, and the original annotation-guided ConvNeXt-Tiny MIL model reached image-level macro-F1 0.922. The corresponding source-image bootstrap intervals for these image-level models are reported in Section 4.3. Full-image YOLO11n reached mAP@0.50 of 0.175, while the higher-capacity YOLO11s control reached 0.170. Because the task values use different prediction units, Table 7 is an orientation summary rather than a single cross-task statistical ranking.

### 4.2. Full-Image and Crop-Based Baselines

ConvNeXt-Tiny had the highest thresholded full-image macro-F1, whereas ResNet50 had the highest macro-AUROC and macro average precision. The difference shows that ranking performance and thresholded multi-label decisions need not identify the same model. The full-image results are reported in Table 8.

**Figure 2:**
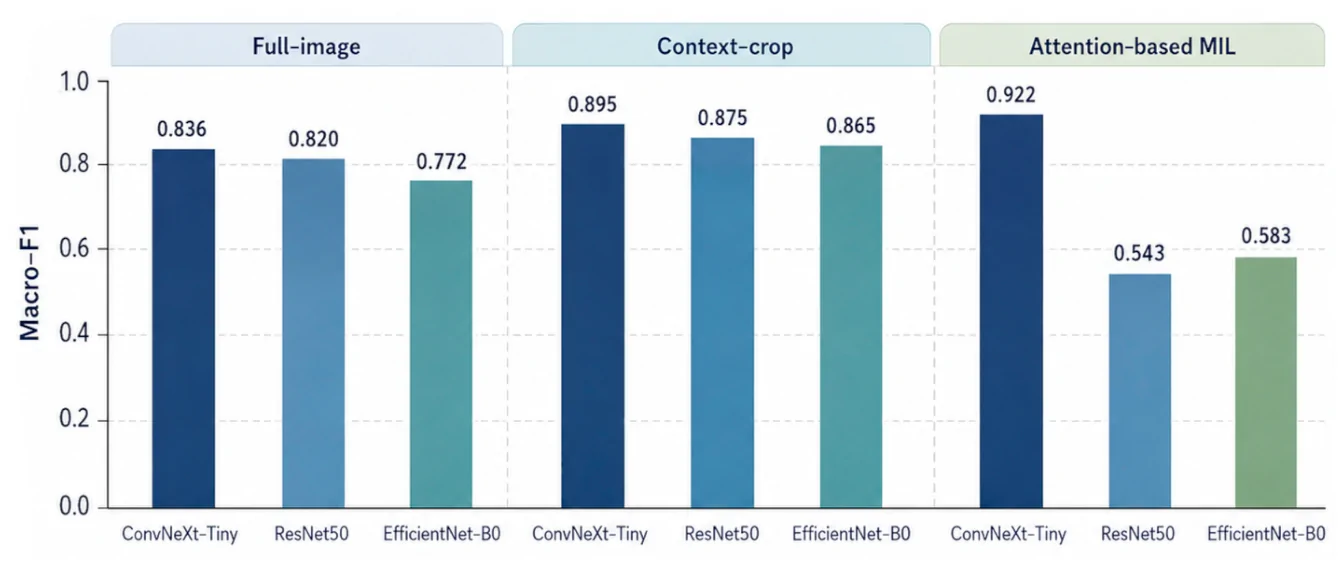
Task-specific macro-F1 values by prediction unit.

At a regional level, context-crop ConvNeXt-Tiny achieved crop macro-F1 0.895. However, its maximum aggregation produced macro-F1 0.799 at image level, below the context-crop ResNet50 value of 0.869. Thus, the most accurate local classifier was not the best fixed-aggregation image classifier. Table 9 reports the crop-level and aggregated image-level results, and Figure 2 groups task-specific values by prediction unit.

### 4.3. Annotation-Guided MIL and Performance by Class

The original context-crop ConvNeXt-Tiny MIL model reached macro-F1 0.922, micro-F1 0.889, exact-match accuracy 0.781, macro-AUROC 0.968, and macro average precision 0.968. EfficientNet-B0 and ResNet50 were weaker in the same aggregation framework; Table 10 reports the encoder comparison. The controlled ablations use a contemporaneous reproduction run rather than replacing the original reported model; the reproduction macro-F1 was 0.912, a difference of −0.0106 from the original and within the pre-specified ±0.02 gate.

Source-image bootstrap intervals for the primary image-level models are reported in Table 11.

For the pre-specified primary macro-F1 comparisons, MIL exceeded full-image ConvNeXt-Tiny by 0.0866 (95% paired-bootstrap CI 0.0285–0.1634; randomization *p* = 0.0064; Holm-adjusted *p* = 0.0128) and context-crop ResNet50 aggregation by 0.0534 (0.0046–0.1141; *p* = 0.0356; adjusted *p* = 0.0356). Secondary results were less uniform: the MIL-versus-crop differences for micro-F1 (*p* = 0.0596) and exact match (*p* = 0.0525) did not cross the 0.05 threshold. These results quantify prediction differences on the fixed 73-image test set rather than multi-split stability.

**Figure 3:**
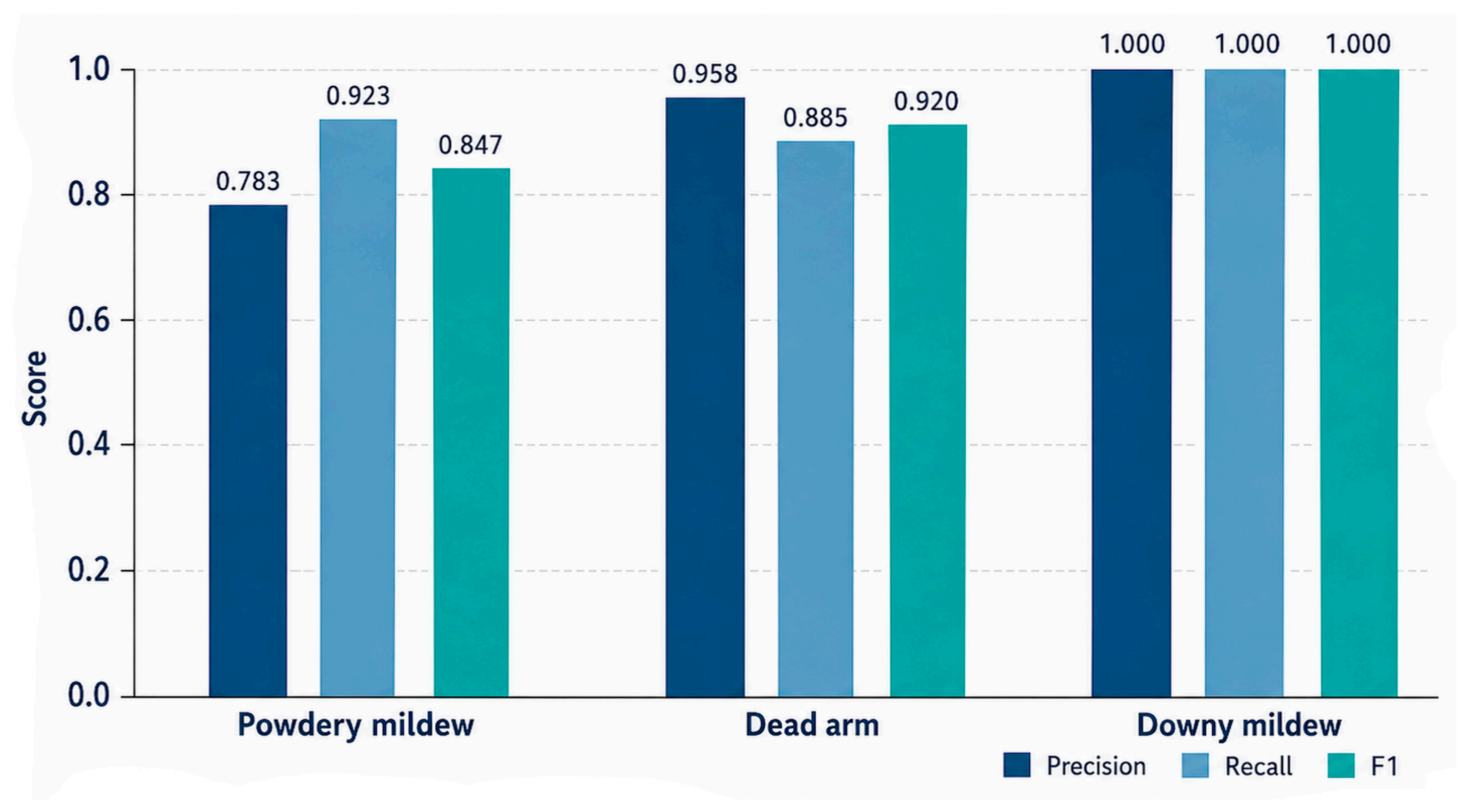
Class-wise precision, recall, and F1 for the best MIL model.

The class-wise results clarify the macro average. For powdery mildew, MIL reached a recall of 0.923 and a precision of 0.783. Dead arm reached precision 0.958 and F1 0.920. Downy mildew reached precision, recall, and F1 of 1.000 on nine positive test images; for both precision and recall, the exact 95% Clopper-Pearson interval was 0.664–1.000. The broad interval shows that the perfect point estimate remains uncertain despite the 212 local downy-mildew boxes contained in those nine source images. Table 12 reports class-wise metrics and image-level support, and Figure 3 visualizes the original MIL point estimates.

### 4.4. Controlled MIL Ablations

Table 13 summarizes the controlled ConvNeXt-Tiny MIL ablation experiments conducted on the fixed source-image split. The contemporaneous full-model rerun reached a macro-F1 of 0.912 (95% CI: 0.868–0.948), a micro-F1 of 0.883, and an exact-match accuracy of 0.767. Replacing gated attention with uniform mean pooling lowered the macro-F1 point estimate to 0.860, whereas removing the auxiliary instance loss lowered it to 0.862. In contrast, replacing the context crops with the original bounding-box crops produced a macro-F1 of 0.915. As reported in Table 13, the paired confidence intervals for all three reference-minus-ablation differences included zero, and none of the three pre-specified macro-F1 comparisons remained statistically significant after Holm correction. Thus, the observed point estimates are compatible with potentially beneficial trends for learned attention and auxiliary regional supervision, but they do not establish statistically independent component effects. Likewise, the evaluated 1.75x context expansion was not supported as a performance-improving component under the present experimental conditions.

### 4.5. Bag Errors and Attention Concentration

**Figure 4:**
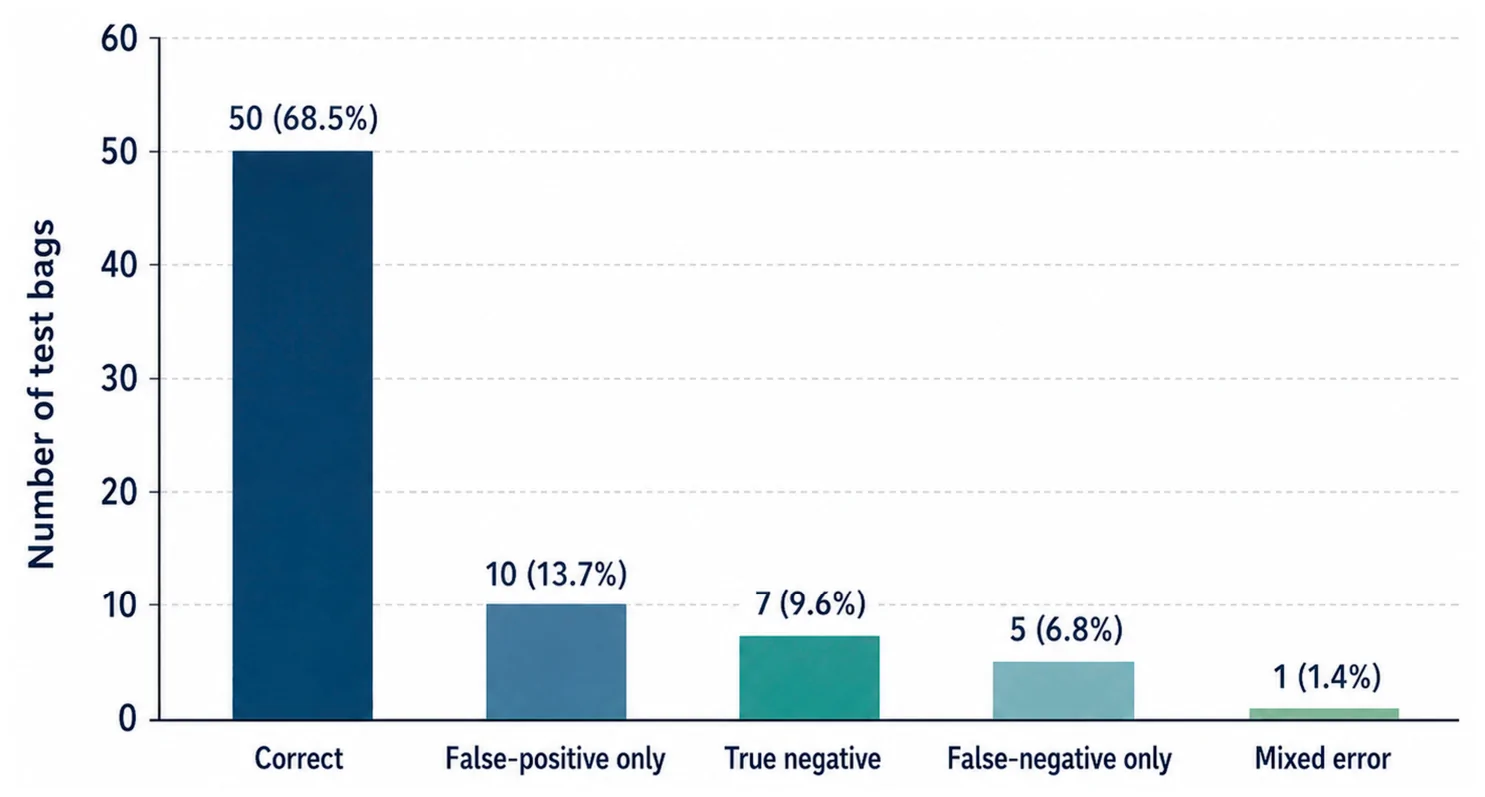
Bag-level error categories for the best MIL model.

Of the 73 original-model test bags, 50 were fully correct and seven were true negatives. The most frequent non-perfect category was false-positive-only prediction in 10 bags. Five bags contained only false negatives, and one contained both an extra and a missed label. Table 14 summarizes the original-model error profile and attention statistics, while Figure 4 shows the bag-level error categories.

**Figure 5:**
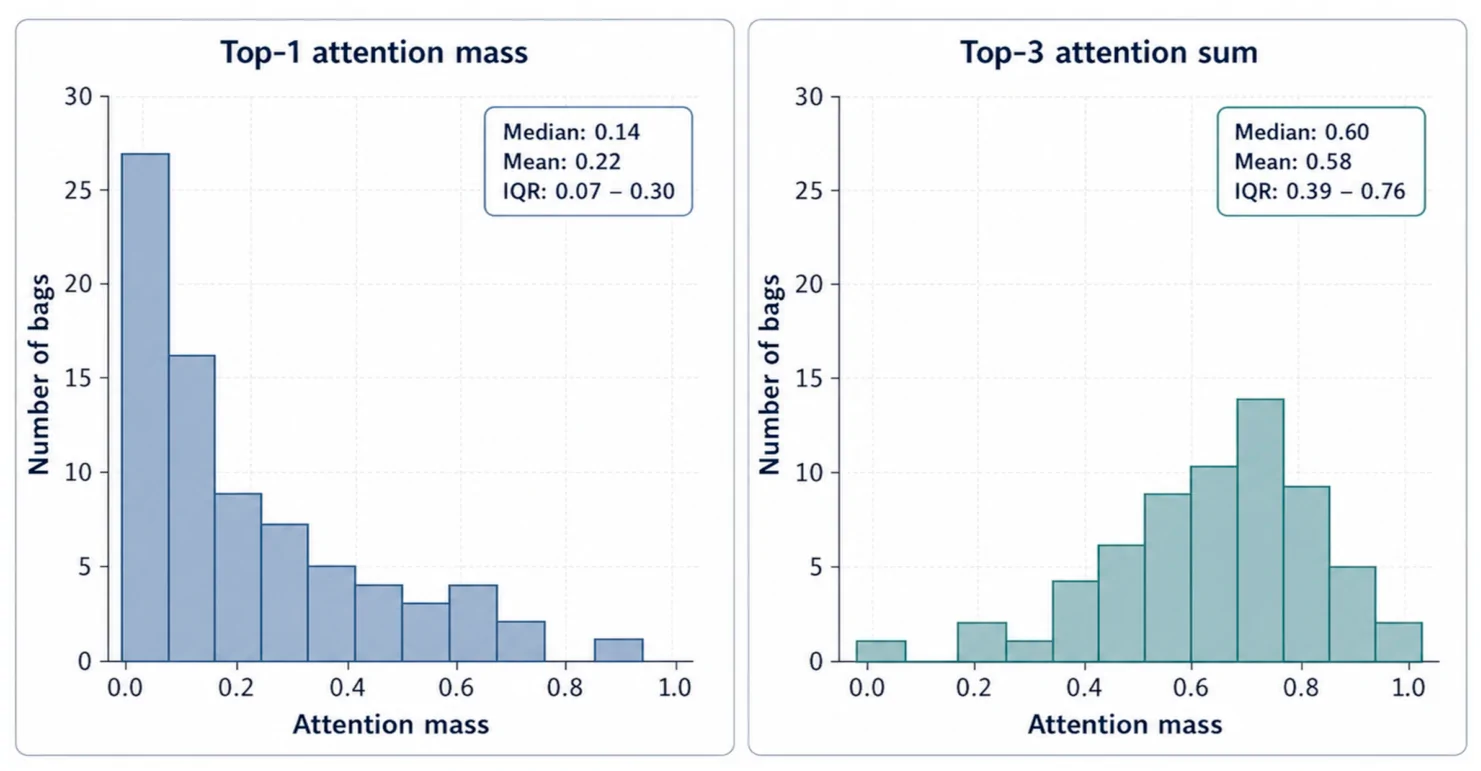
Top-1 and top-3 attention mass across test bags.

**Figure 6:**
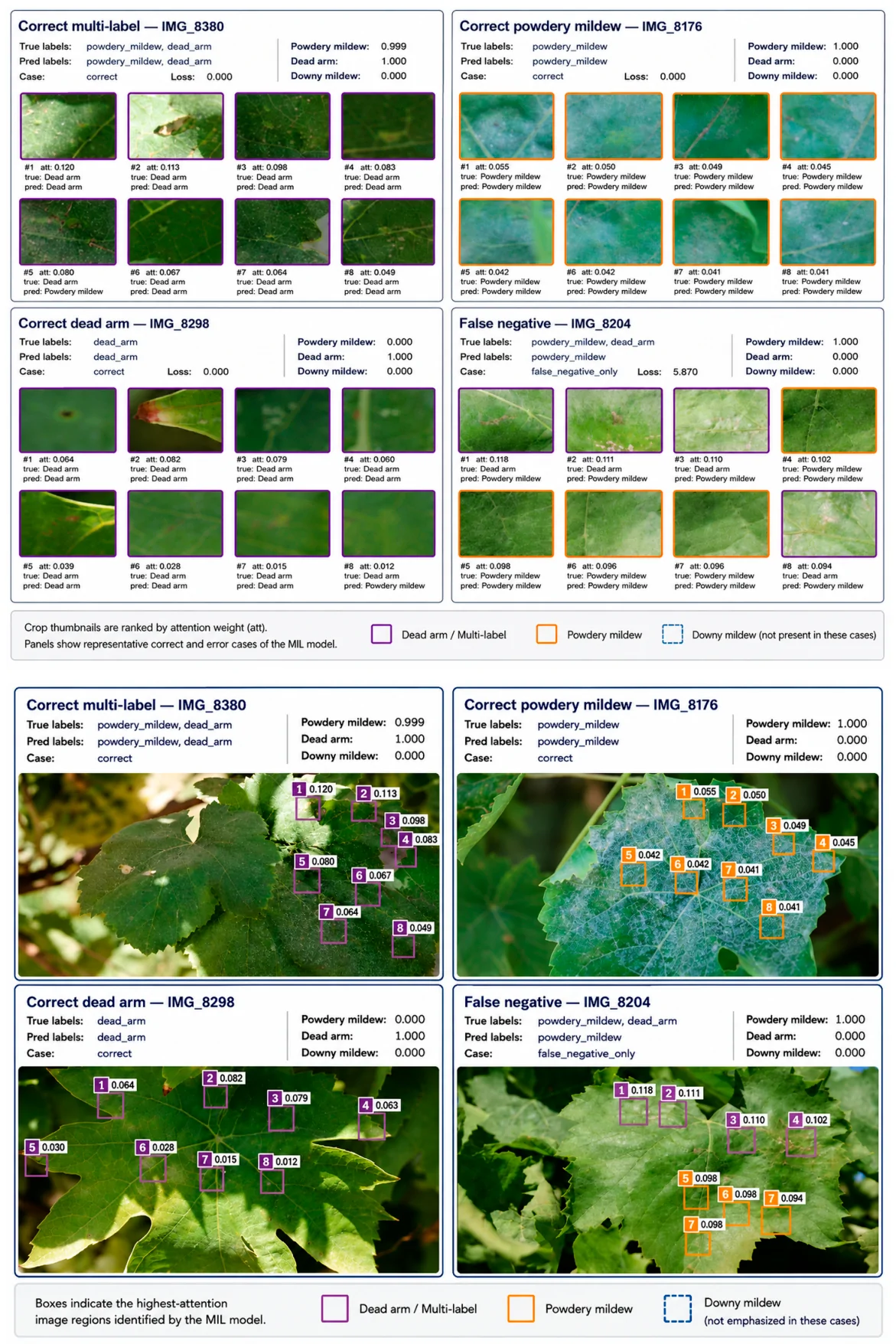
Qualitative attention evidence from ranked crops and source image overlays.

Figure 5 summarizes the distribution of top-1 and top-3 attention mass across the test bags. Figure 6 provides complementary qualitative evidence by pairing attention ranked context crops with source image overlays for representative MIL predictions. The panels are used for visual audit only: attention is shared across disease labels, raw entropy is unnormalized, and no quantitative overlap-based localization score was available.

### 4.6. YOLO11 Localization and Capacity Control

**Figure 7:**
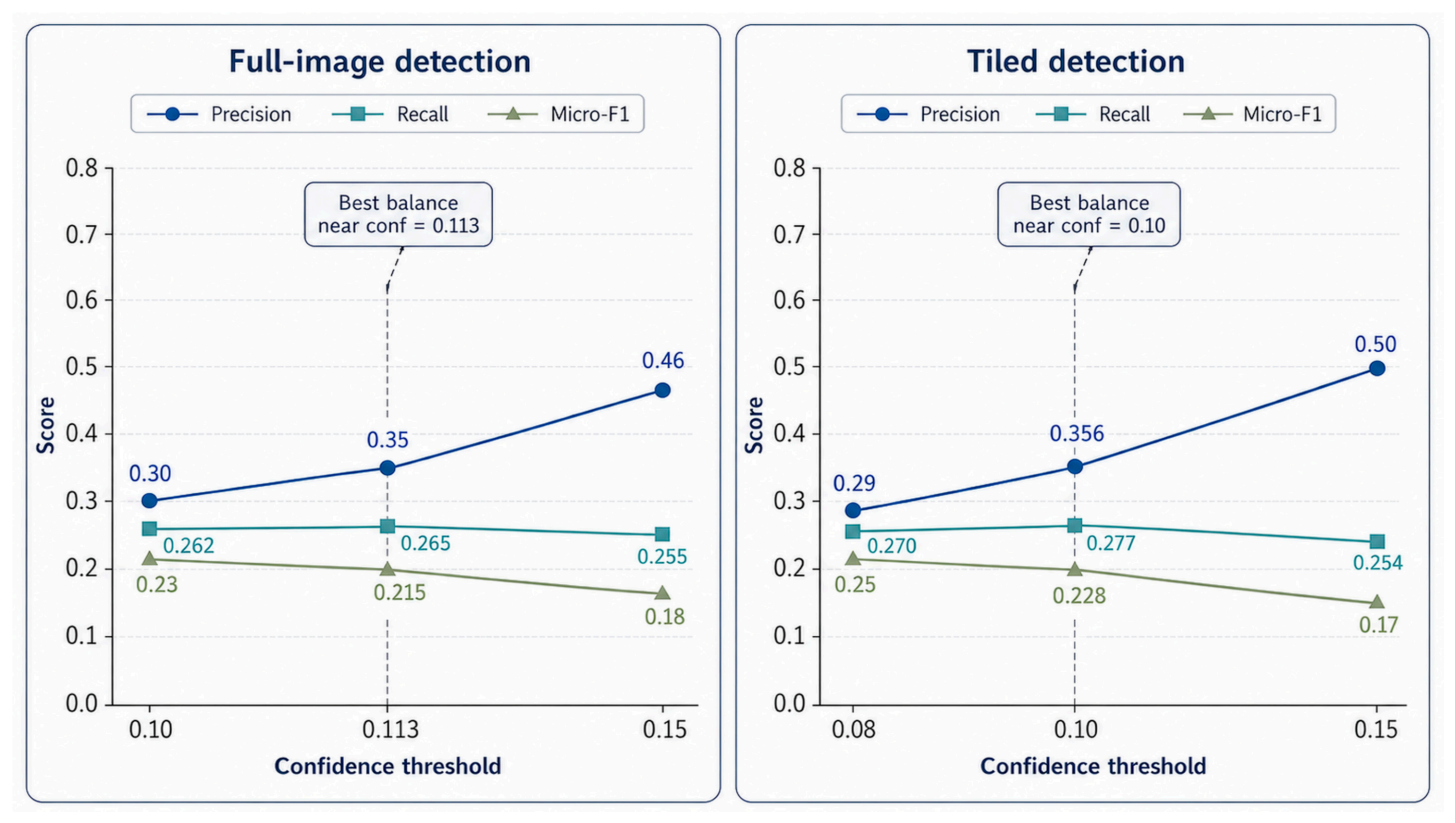
Detection performance across confidence thresholds.

**Figure 8:**
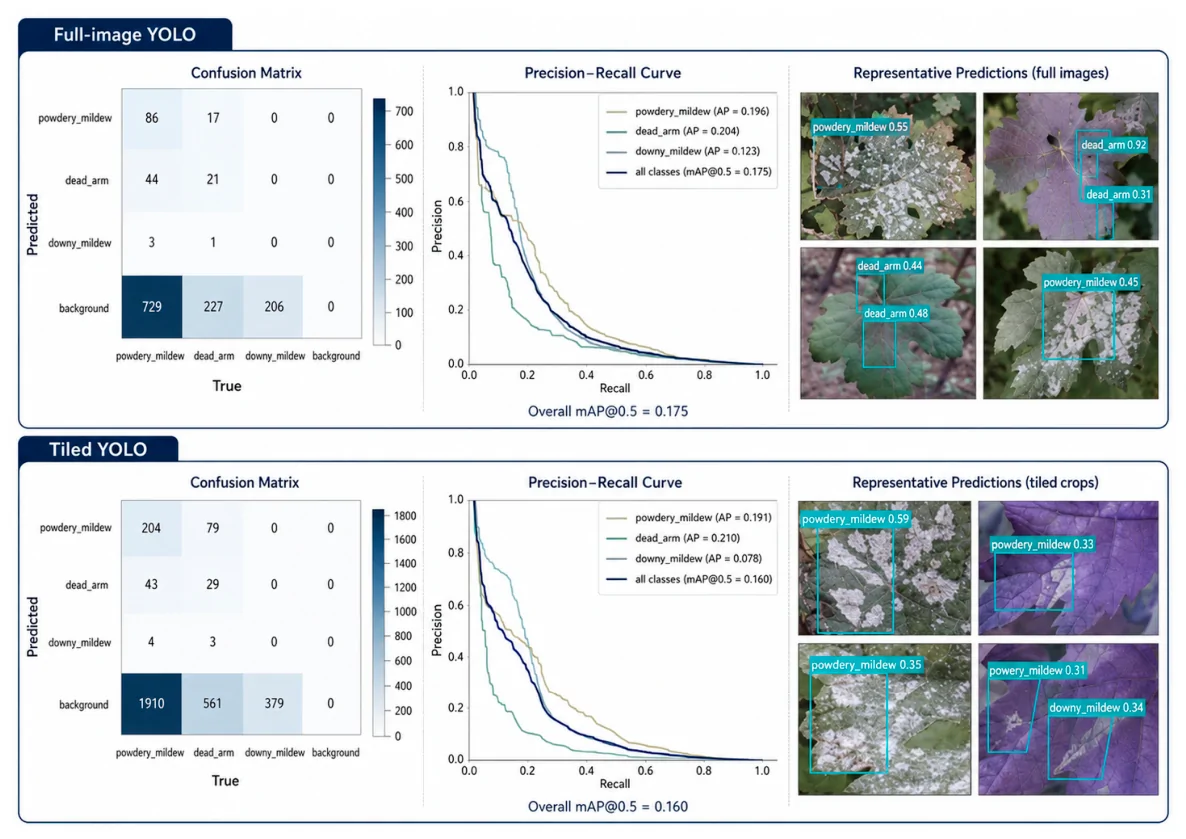
Full-image and tiled YOLO11n comparison; the YOLO11s capacity control is reported in Table 15.

The full-image YOLO11n configuration reached precision 0.330, recall 0.192, mAP@0.50 0.175, and mAP@0.50:0.95 0.0569. The higher-capacity full-image YOLO11s control, trained under the same protocol, reached precision 0.286, recall 0.202, mAP@0.50 0.170, and mAP@0.50:0.95 0.0514. Thus, increasing capacity from the nano to the small variant did not improve the evaluated full-image localization metrics. The tiled YOLO11n configuration reached mAP@0.50 0.160 and slightly higher recall than full-image YOLO11n. In the descriptive YOLO11n threshold sweep, the highest observed micro-F1 was 0.265 for full images and 0.276 for tiles. Table 15 separates standard detector metrics from post hoc threshold results; Figure 7 shows the threshold sweep, and Figure 8 shows the full-image versus tiled YOLO11n diagnostics.

## 5. Discussion

This section interprets the fixed-split uncertainty analysis, component ablations, detector capacity control, class support, and model-derived evidence within the annotation-assisted and strictly offline scope of the study.

The first point concerns how known local evidence is organized for image-level recognition. The original annotation-guided ConvNeXt-Tiny MIL model achieved macro-F1 0.922 with a source-image bootstrap interval of 0.882–0.956. On the same 73 test images, paired macro-F1 differences relative to the full-image and maximum-aggregation baselines were positive, their paired-bootstrap intervals excluded zero, and the Holm-adjusted randomization *p*-values were 0.0128 and 0.0356. These results provide stronger fixed-test-set evidence than point estimates alone, but they do not establish stability across alternative data partitions, training seeds, or acquisition domains.

The controlled ablations refine the interpretation of the MIL result. Relative to the contemporaneous reproduction reference, mean pooling and removal of the auxiliary instance objective reduced the observed macro-F1 by approximately 0.05, but both paired intervals included zero and the Holm-adjusted tests were not significant. The bounding-box condition slightly exceeded the context-crop reference, with a negligible and non-significant difference. The results therefore do not support attributing the full-model performance to context expansion, and they support only cautious point-estimate trends—not statistically isolated effects—for learned attention and auxiliary regional supervision.

Backbone sensitivity also remains relevant. ConvNeXt-Tiny produced substantially higher original MIL values than EfficientNet-B0 and ResNet50, and the full-model rerun reproduced the original macro-F1 within the pre-specified ±0.02 gate. Nevertheless, a single rerun and fixed split do not establish a stable architectural law. Repeated seeds and grouped multi-split validation would be required to quantify training and partition variability directly.

The detector capacity control narrows the interpretation of the localization result. YOLO11s did not improve full-image mAP@0.50 or mAP@0.50:0.95 relative to YOLO11n under the matched protocol. This indicates that the low localization values were not resolved by this single increase in model capacity, but it does not imply that all larger detectors, two-stage methods, segmentation models, or alternative annotation formulations would behave similarly. The tiled results additionally represent independent tile-level evaluation rather than a remapped and merged source-image detector.

Class support remains an important qualification. The nine positive downy-mildew source images were all classified correctly by the original MIL model, but the exact 95% interval for both precision and recall was 0.664–1.000. The wide interval makes clear why the perfect point estimate is not evidence of stable class-level generalization. Powdery mildew showed high recall with lower precision, while dead arm showed high precision with incomplete recall.

Raw attention entropy and top-k mass provide descriptive views of the observed attention distribution. Because entropy was not normalized for bag size and the attention weights are shared across disease labels, no conclusion is drawn about concentration across bags of different sizes or localization for individual diseases. The selected panels support qualitative visual audit only; quantitative fidelity would require a predefined overlap rule, normalized concentration measures, and class-matched evaluation against ground-truth regions.

Finally, the work is deliberately framed as a retrospective offline study of pre-acquired and manually annotated close-up images. It does not evaluate image-acquisition hardware, real-time operation, mobile or edge execution, latency, FLOPs, memory footprint, parameter efficiency, operational field screening, or performance outside HERMOS. The MIL branch additionally requires manual boxes at inference, so it is an annotation-assisted aggregation benchmark rather than an automatic screening system.

Adaptation to whole-vine imagery would require an upstream stage that detects leaves, canopy regions, or candidate symptomatic areas before local encoding and bag aggregation. Whole-vine views introduce smaller apparent lesions, occlusion, overlapping leaves, background clutter, and a broader scale range. These conditions constitute a distinct domain and annotation problem, and the present results cannot be extrapolated to that setting without dedicated data and external validation.

The scope is therefore defined by annotation-assisted inference, one primary split, fixed-model source-image uncertainty, small image-level support for downy mildew, three single-seed component ablations, two YOLO11 capacities, and qualitative attention analysis. The resulting evidence supports an offline, hypothesis-generating benchmark and does not support deployment or external-generalization claims.

## 6. Conclusions

This retrospective offline study organized dense manual grapevine lesion annotations as local evidence for source-image disease recognition. The original annotation-guided ConvNeXt-Tiny MIL model achieved macro-F1 0.922 (95% source-image bootstrap CI 0.882–0.956), and paired macro-F1 comparisons favored MIL over the full-image and fixed crop-aggregation baselines within the 73-image test set. These intervals and tests quantify fixed-test-sample uncertainty but do not establish multi-split or multi-seed stability. The perfect downy-mildew point estimate was supported by only nine positive source images, with exact precision and recall intervals of 0.664–1.000. Controlled ablations showed lower point estimates without learned attention or auxiliary instance supervision, but no statistically significant independent effects after multiplicity correction; context expansion was not supported as beneficial. YOLO11s did not improve localization over YOLO11n under the matched protocol. The findings support annotation-guided aggregation of pre-specified regions as an offline exploratory formulation, not an automatic, efficient, mobile, or externally validated deployment system.

## Figures and Tables

**Table 1:** Dataset characteristics after local audit.

Metric	Value	Interpretation
Image files/XML files	492/492	Paired images and Pascal VOC annotations
Parsed objects	13,904	Objects parsed before validity filtering
Usable canonical objects	13,765	After label mapping and invalid box filtering
Retained source images	491	One image excluded because no usable object remained
Canonical regional classes	4	Healthy, powdery mildew, dead arm, downy mildew
Images with >1 canonical regional class	181	Regional-class coexistence; not necessarily multiple disease presence labels
Mean objects per image	28.03	Dense regional annotation setting
Maximum objects per image	105	High within-image object density
Median box area/image area	0.00229	Small regional scale

**Table 2:** Comparison with widely used plant disease datasets.

Dataset	Images	Categories	Annotation	Acquisition and Sensing Relevance
PlantVillage [17]	54,306	38 crop disease labels	Image-level	Mostly controlled backgrounds; broad classification benchmark
PlantDoc [18]	2598	27 categories	Object boxes	Natural/field images; multi-crop disease detection
HERMOS [16]	492 (491 used)	4 regional classes	Dense Pascal VOC boxes	Field-oriented grapevine images; multiple local regions per source image

**Table 3:** Source image split and regional object distribution.

Split	Images	Images with >1 Regional Class	Objects	Healthy	Powdery Mildew	Dead Arm	Downy Mildew
Train	344	127	9651	3542	3808	1306	995
Validation	74	27	2069	755	821	286	207
Test	73	27	2045	747	815	271	212

**Table 4:** Learning formulations and evaluation targets.

Formulation	Input Representation	Prediction Target	Primary Metric	Purpose
Full image classification	Whole source image	Three disease presence labels	Macro-F1	Image-level diagnosis without regional cropping
Crop classification	Bounding box or context crop	Four regional classes	Crop macro-F1	Local regional discriminability
Crop-to-image aggregation	Maximum crop probabilities per image	Three disease presence labels	Macro-F1	Fixed heuristic aggregation
Annotation-guided MIL	Manual context crops as a source image bag	Three disease presence labels	Macro-F1	Learned aggregation of annotation-derived local evidence
YOLO11 detection	Full images or overlapping tiles; YOLO11n primary and YOLO11s capacity control	Three disease box classes	mAP@0.50	Strict-localization baselines under the offline protocol

**Table 5:** Exact preprocessing, training, clipping, validation-selection, and statistical-analysis settings.

Component	Item	Exact Value or Rule
Shared protocol	Split and random seed	Source-image split 344/74/73 (train/validation/test); seed 52; all derived views inherited the source-image assignment.
Archived environment	Software and device	Python 3.13.5; PyTorch 2.11.0; torchvision 0.26.0; timm 1.0.30; Ultralytics 8.4.26. Classification/MIL used Apple MPS with AMP ineffective/disabled; YOLO used CPU with deterministic = true.
Crop export	Bounding-box crop	Valid manual box clipped to the image; exported to 150 × 150 JPEG, quality 95, Lanczos resampling.
Crop export	Context crop geometry	Square centered on the manual box; side = 1.75 × max (width, height); lower coordinates floor, upper coordinates ceil; clipped to image boundaries; no padding.
Full-image models	Input and augmentation	384 × 384 bicubic resize; ImageNet mean/std. Training: horizontal flip 0.5, vertical flip 0.25, rotation ±15 degrees, color jitter brightness/contrast 0.15, saturation 0.10, hue 0.03.
Full-image models	Loss and weights	BCEWithLogitsLoss; positive weights [0.890, 1.774, 7.390] for powdery mildew, dead arm, and downy mildew.
Full-image models	Optimization	AdamW; learning rate 1 × 10^−4^; weight decay 1 × 10^−4^; batch 8; max 40 epochs; gradient clip 1.0; ReduceLROnPlateau on validation loss, factor 0.5, patience 4.
Full-image models	Checkpoint and threshold rule	Best checkpoint by validation macro-F1 at fixed 0.5 thresholds; early-stopping patience 8, min_delta 10^−4^. Final per-class thresholds selected on validation by 0.01 F1 grid and frozen for test; primary ConvNeXt-Tiny thresholds [0.53, 0.44, 0.22].
Crop classifiers	Input and augmentation	224 × 224 bilinear resize; ImageNet mean/std. Training: horizontal flip 0.5, vertical flip 0.5, rotation ±15 degrees, brightness/contrast 0.15, saturation 0.10.
Crop classifiers	Loss, optimization, selection	Class-weighted cross-entropy; weights [0.681, 0.634, 1.847, 2.425]; no balanced sampler; AdamW 10^−4^, weight decay 10^−4^; batch 32; max 35 epochs; clip 1.0; ReduceLROnPlateau on validation crop macro-F1 (factor 0.5, patience 2, min_lr 10^−7^); early patience 7, min_delta 10^−5^.
Crop aggregation	Aggregation and thresholds	Maximum disease probability over crops per source image; validation 0.01 F1 grid, then frozen for test. Primary context-ResNet50 thresholds [0.40, 0.89, 0.90].
MIL	Architecture and initialization	ConvNeXt-Tiny encoder initialized from the context-crop classifier checkpoint (epoch 15); gated attention dimension 256; dropout 0.2; all instances retained; bag batch size 2; 224 × 224 input.
MIL	Losses and weights	Bag weighted BCEWithLogitsLoss [1.000, 1.774, 7.390] plus lambda x instance cross-entropy [0.681, 0.634, 1.847, 2.425]; lambda = 0.2.
MIL	Optimization and selection	AdamW 1 × 10^−4^, weight decay 1 × 10^−4^; max 40 epochs; gradient clip 1.0; ReduceLROnPlateau on validation macro-F1 (factor 0.5, patience 3); early patience 8, min_delta 10^−5^. Validation thresholds optimized each epoch; best checkpoint by validation macro-F1. Original best epoch 17 of 25.
MIL	Final thresholds	Powdery mildew 0.14; dead arm 0.29; downy mildew 0.05; selected on validation and frozen for test.
YOLO11n/11s	Training schedule	Pretrained weights; input 1280; max 80 epochs; batch 4; workers 4; optimizer auto; lr0 0.01, lrf 0.01, momentum 0.937, weight decay 5 × 10^−4^; warmup 3 epochs; close mosaic 10; patience 15; seed 52.
YOLO11n/11s	Augmentation	HSV h/s/v = 0.015/0.7/0.4; translate 0.1; scale 0.5; horizontal flip 0.5; mosaic 1.0; degrees, shear, perspective, vertical flip, mixup, cutmix, and copy-paste = 0.
YOLO tiled view	Tiling and clipping	Tile 1280 × 1280; overlap 0.25; stride 960; minimum visible-box fraction 0.25; minimum clipped width/height 4 pixels; negative-tile ratio 0.25; tiles evaluated independently without source-image remapping/merging.
YOLO validation	NMS and checkpoint	IoU 0.70; max_det 300; class-aware NMS; framework-default confidence for standard validation; best.pt by validation fitness (mAP@0.50:0.95).
Statistics	Uncertainty and paired tests	10,000 source-image bootstrap replicates with percentile 95% intervals; 10,000 paired prediction-swap randomizations for macro-/micro-F1; exact McNemar for exact match; Holm adjustment within each pre-specified macro-F1 family; exact Clopper-Pearson intervals for all-success downy-mildew precision/recall.

**Table 6:** Input tensor shapes and exact trainable parameter counts for the evaluated task-specific architectures.

Formulation	Backbone/Model	Model Input Shape	Trainable Parameters
Full-image multi-label classification	ResNet50	3 × 384 × 384	23,514,179 (23.51 M)
	EfficientNet-B0	3 × 384 × 384	4,011,391 (4.01 M)
	ConvNeXt-Tiny	3 × 384 × 384	27,822,435 (27.82 M)
Regional crop classification (bounding-box and context views)	ResNet50	3 × 224 × 224	23,516,228 (23.52 M)
	EfficientNet-B0	3 × 224 × 224	4,012,672 (4.01 M)
	ConvNeXt-Tiny	3 × 224 × 224	27,823,204 (27.82 M)
Annotation-guided gated-attention MIL	ResNet50	n_i_ × 3 × 224 × 224 per bag	24,571,720 (24.57 M)
	EfficientNet-B0	n_i_ × 3 × 224 × 224 per bag	4,672,644 (4.67 M)
	ConvNeXt-Tiny	n_i_ × 3 × 224 × 224 per bag	28,219,496 (28.22 M)
Three-class lesion detection	YOLO11n	3 × 1280 × 1280	2,590,409 (2.59 M)
	YOLO11s	3 × 1280 × 1280	9,428,937 (9.43 M)

Note: The batch dimension is omitted for single-image models. For MIL, *n_i_* denotes the variable number of manually annotated regional instances in source-image bag *i*. Counts include all task-specific trainable layers; the MIL counts include the shared encoder, gated-attention module, bag classifier, and auxiliary instance classifier. Detector input shapes refer to tensors after resizing/letterboxing. Non-trainable buffers and optimizer states are excluded.

**Table 7:** Cross-task summary of the fixed-split results.

Task	Highest Observed Setting	Value	Metric	Interpretation
Full image disease presence	ConvNeXt-Tiny	0.836	Macro-F1	Whole-image baseline
Crop classification	Context crop ConvNeXt-Tiny	0.895	Crop macro-F1	Strong local recognition
Crop-to-image aggregation	Context crop ResNet50	0.869	Image macro-F1	Fixed maximum aggregation
Annotation-guided MIL	Context crop ConvNeXt-Tiny	0.922	Macro-F1	Highest original image-level value; fixed-split CI reported in Section 4.3
Lesion detection	Full-image YOLO11n	0.175	mAP@0.50	Best detector result; YOLO11s capacity control reported in Section 4.6

**Table 8:** Full-image multi-label results.

Model	Macro-F1	Micro-F1	Exact Match	Macro-AUROC	Macro-AP
ConvNeXt-Tiny	0.836	0.808	0.658	0.919	0.900
ResNet50	0.820	0.814	0.685	0.941	0.920
EfficientNet-B0	0.772	0.753	0.616	0.896	0.863

**Table 9:** Crop classification and crop-to-image aggregation results.

View	Model	Macro-F1		Micro-F1	Exact Match
		Crop F1	Image F1		
Context	ResNet50	0.875	0.869	0.838	0.644
Bounding box	EfficientNet-B0	0.888	0.835	0.848	0.699
Bounding box	ResNet50	0.857	0.835	0.833	0.671
Context	EfficientNet-B0	0.865	0.832	0.846	0.726
Context	ConvNeXt-Tiny	0.895	0.799	0.789	0.562
Bounding box	ConvNeXt-Tiny	0.890	0.764	0.805	0.589

**Table 10:** Annotation-guided MIL encoder results.

Encoder	Macro-F1	Micro-F1	Exact Match	Macro-AUROC	Macro-AP
ConvNeXt-Tiny	0.922	0.889	0.781	0.968	0.968
EfficientNet-B0	0.583	0.601	0.247	0.729	0.603
ResNet50	0.543	0.607	0.205	0.616	0.474

**Table 11:** Source-image bootstrap uncertainty for the primary image-level models.

Model	Macro-F1 (95% CI)	Micro-F1 (95% CI)	Exact Match (95% CI)
Full-image ConvNeXt-Tiny	0.836 (0.754–0.895)	0.808 (0.737–0.871)	0.658 (0.548–0.767)
Context-crop ResNet50 maximum aggregation	0.869 (0.804–0.917)	0.838 (0.783–0.889)	0.644 (0.534–0.753)
Annotation-guided ConvNeXt-Tiny MIL	0.922 (0.882–0.956)	0.889 (0.838–0.935)	0.781 (0.685–0.877)

**Table 12:** Image-level results and test support by class.

Task	Class	F1	Precision	Recall	Positive Test Images
Full image	Powdery mildew	0.829	0.791	0.872	39
Full image	Dead arm	0.737	0.677	0.808	26
Full image	Downy mildew	0.941	1.000	0.889	9
Annotation-guided MIL	Powdery mildew	0.847	0.783	0.923	39
Annotation-guided MIL	Dead arm	0.920	0.958	0.885	26
Annotation-guided MIL	Downy mildew	1.000	1.000	1.000	9

**Table 13:** Controlled ConvNeXt-Tiny MIL ablations on the fixed source-image split.

Condition	Macro-F1 (95% CI)	Micro-F1	Exact Match	Reference-Condition: Macro-F1 Difference (95% CI); Holm p
Full-model rerun: context, gated attention, lambda = 0.2	0.912 (0.868–0.948)	0.883	0.767	Reference
No learned attention: uniform mean pooling	0.860 (0.761–0.930)	0.851	0.753	+0.051 (−0.015 to 0.150); 0.517
No context expansion: bounding-box crops, same initialization	0.915 (0.844–0.962)	0.892	0.795	−0.003 (−0.052 to 0.066); 0.910
No auxiliary instance loss: lambda = 0	0.862 (0.778–0.926)	0.847	0.740	+0.050 (−0.018 to 0.140); 0.517

**Table 14:** MIL error profile and attention summary.

Item	Value	Interpretation
Fully correct bags	50	All disease labels matched
True negatives	7	No disease predicted and no disease present
False-positive only	10	At least one extra disease label
False-negative only	5	At least one true disease label missed
Mixed error	1	Both an extra and a missed label
Attention entropy, mean	2.69	Raw, unnormalized entropy; affected by bag size
Attention entropy, median	2.82	Raw, unnormalized entropy; affected by bag size

**Table 15:** YOLO11 detection, capacity control, and fixed-threshold results.

View/Setting	Precision	Recall	mAP@0.50 or F1	mAP@0.50:0.95/Note
Full-image YOLO11n	0.330	0.192	0.175	0.0569
Tiled YOLO11n	0.294	0.201	0.160	0.0499
Full-image YOLO11s	0.286	0.202	0.170	0.0514; higher-capacity control
Full YOLO11n, conf = 0.113	0.344	0.215	0.265	Highest observed micro-F1 in reported sweep
Tiled YOLO11n, conf = 0.100	0.355	0.226	0.276	Highest observed micro-F1 in reported sweep

## Data Availability

The HERMOS dataset is publicly available in Mendeley Data (Version 2; https://doi.org/10.17632/j4xs3kh3fd.2) under the Creative Commons Attribution 4.0 International (CC BY 4.0) license. All HERMOS-derived image examples in this article are used with attribution under that license. Derived manifests, source-image split definitions, archived run configurations, per-source prediction tables, bootstrap and paired-test outputs, and ablation/capacity-control summaries are available from the corresponding author on reasonable request, subject to repository and project constraints. HERMOS source-image content shown in Figure 1, Figure 6, and Figure 8 is used under the same CC BY 4.0 license; the workflow graphics, predictions, overlays, and layouts are original. High-resolution versions of Figure 1, Figure 6, and Figure 8 are supplied separately.
